# Annihilation of Excess Excitations along Phycocyanin Rods Precedes Downhill Flow to Allophycocyanin Cores in the Phycobilisome of *Synechococcus elongatus* PCC 7942

**DOI:** 10.1021/acs.jpcb.1c06509

**Published:** 2022-01-04

**Authors:** Polina Navotnaya, Siddhartha Sohoni, Lawson T. Lloyd, Sami M. Abdulhadi, Po-Chieh Ting, Jacob S. Higgins, Gregory S. Engel

**Affiliations:** Department of Chemistry, James Franck Institute and Institute for Biophysical Dynamics, The University of Chicago, Chicago, Illinois 60637, United States

## Abstract

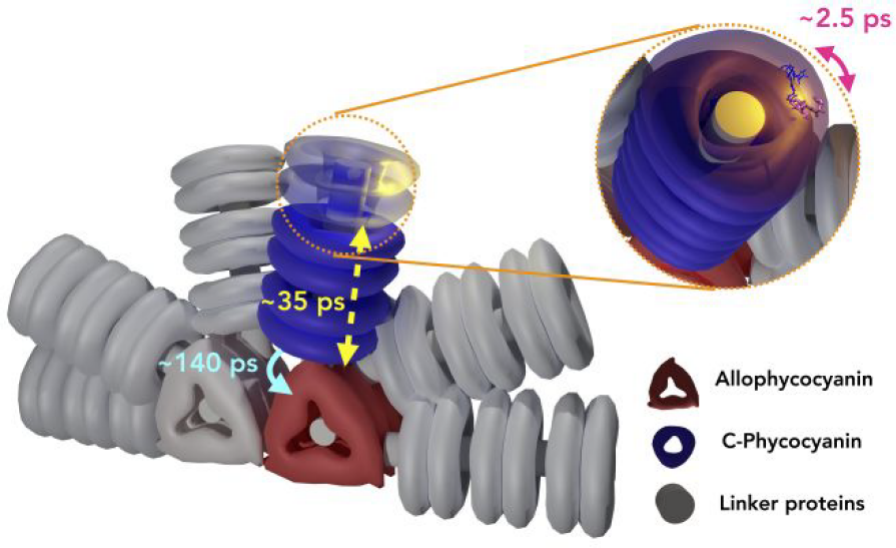

Cyanobacterial phycobilisome complexes absorb visible sunlight and funnel photogenerated excitons to the photosystems where charge separation occurs. In the phycobilisome complex of *Synechococcus elongatus* PCC 7942, phycocyanin protein rods that absorb bluer wavelengths are assembled on allophycocyanin cores that absorb redder wavelengths. This arrangement creates a natural energy gradient toward the reaction centers of the photosystems. Here, we employ broadband pump–probe spectroscopy to observe the fate of excess excitations in the phycobilisome complex of this organism. We show that excess excitons are quenched through exciton–exciton annihilation along the phycocyanin rods prior to transfer to the allophycocyanin cores. Our observations are especially relevant in comparison to other antenna proteins, where exciton annihilation primarily occurs in the lowest-energy chlorophylls. The observed effect could play a limited photoprotective role in physiological light fluences. The exciton decay dynamics is faster in the intact phycobilisome than in isolated C-phycocyanin trimers studied in earlier work, confirming that this effect is an emergent property of the complex assembly. Using the obtained annihilation data, we calculate exciton hopping times of 2.2–6.4 ps in the phycocyanin rods. This value agrees with earlier FRET calculations of exciton hopping times along phycocyanin hexamers by Sauer and Scheer.

## Introduction

Cyanobacteria carry out nitrogen fixation, methanogenesis, and oxygenic photosynthesis in the biosphere.^1^ The efficiency with which a photogenerated exciton reaches the reaction center, or the transfer-to-trap quantum efficiency, is remarkably high in these organisms, with values reported in the range of 80–95%.^2,3^ An energetic funnel in which photogenerated excitons move downhill from light-harvesting antennae to the reaction center is responsible for this high transfer-to-trap efficiency.^3,4^

The antenna complexes of cyanobacterial photosynthesis strongly absorb all visible wavelengths shorter than 700 nm. Light between 550 and 700 nm is absorbed by tetrapyrrole-based phycobilin (PB) chromophores which are found in the antenna complex assembly known as the phycobilisome (PBS). The PBS complex attaches to the stromal side of the membrane and funnels photogenerated excitons to both Photosystem I (PSI) and Photosystem II (PSII).^5^

**Figure 1:**
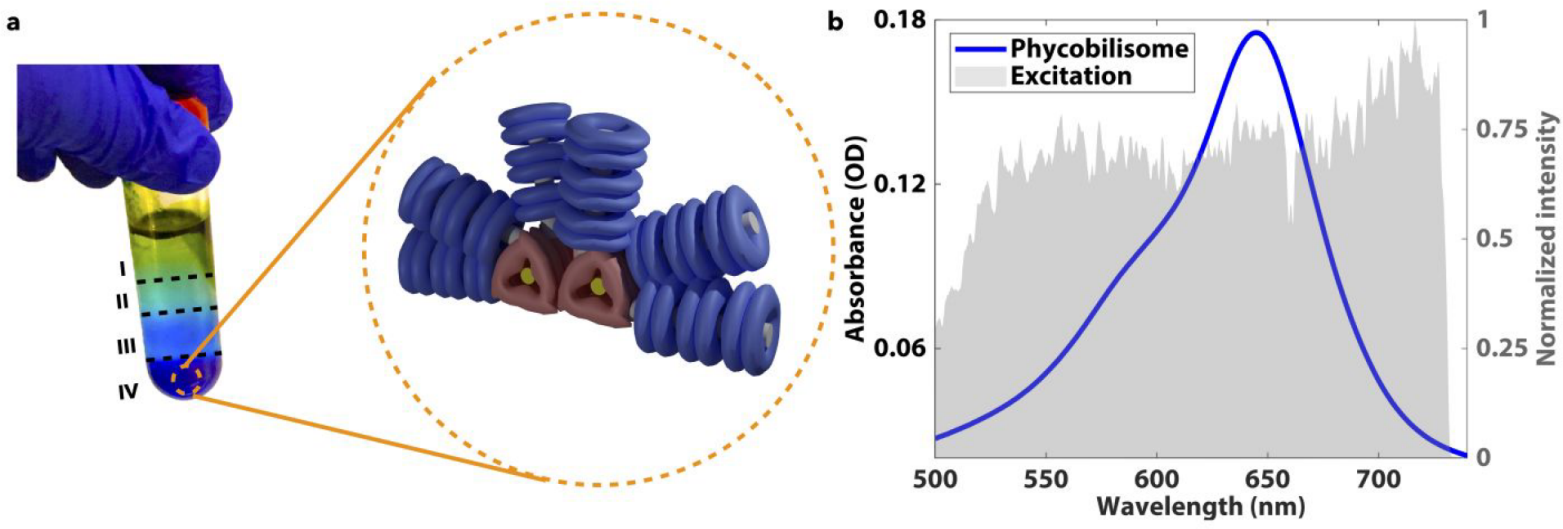
(a) Phycobilisome isolated in the 1.5 M sucrose layer of the sucrose gradient and the structure of the *Synechococcus el.* PCC 7942 phycobilisome. The phycocyanin rods are shown in blue, and the allophycocyanin core is shown in red. (The image contrast of the centrifuge tube photograph has been adjusted for clarity; the raw image is included in Supporting Information Figure 7 for reference). Linker proteins are shown in green and gray. (b) Absorption spectrum of the phycobilisome and the laser spectrum used in this study.

In the PBS complex, phycobilin chromophores are covalently bound to the water-soluble phycobiliproteins.^6−8^ The PBS structure of *S. elongatus* PCC 7942 is shown in Figure 1 along with its absorption spectrum. This PBS complex is made of six rods that are attached to a core. Each rod consists of three hexamers of the phycocyanin (PC) protein. Each PC monomer binds three phycocyanobilin molecules,^9−11^ and the core consists of two tetramers of four trimers of allophycocyanin (APC). The rods are connected to the core by linker proteins. Phycocyanobilin is the only phycobilin chromophore found in *S. elongatus* PCC 7942. Previous studies have shown that interchromophore delocalization of excitations is seen on the subpicosecond time scales in APC but not in PC.^12−15^ Chromophore protein environments combined with chromophore–chromophore interactions in APC tune the absorption of PC to ∼620 nm and that of APC to ∼650 nm to maintain the energetic funnel.^11^ On the picosecond–nanosecond time scale, FRET hopping can be used to accurately describe exciton transport in the phycobilisome.^16^

The redox chemistry of the PSI and PSII reaction centers (RCs) is typically slower than the photon absorption rate of the antennas under high solar-fluence conditions.^4,17^ In clear water under sunny conditions, caustics can form which strongly focus sunlight, creating millisecond transients in shallow water. These transients can momentarily create solar fluxes 5–10 times the typical intensity at the surface of the water, also contributing to the large imbalance between the photon absorption rate and the reaction center turnover rate.^18,19^ In such scenarios, numerous photoprotective strategies are employed to dissipate excess excitations and avoid stressing the RC or the production of singlet oxygen species. For example, PSI RCs are surrounded by red chlorophylls, which absorb wavelengths redder than 700 nm and hold funneled excitons to slow the exciton trapping process in the reaction center, suggesting a photoprotective role of these chlorophylls.^20^ In numerous antenna complexes, notably LH2 and LHCII, exciton annihilation also occurs predominantly in the lowest-energy chromophores before excitations are passed on toward the antenna.^2,21,22^ This phenomenon could play a photoprotective role. In *Synechocystis sp*. PCC 6803, a small light-activated protein called the orange carotenoid protein (OCP) binds to the PBS complex to drive the nonphotochemical quenching of excess excitations^11,23−25^ prior to reaching the reaction center. Intrinsic light-activated dissipation has also been shown in this species.^26^ In *S. elongatus*, the formation of IsiA-PSI supercomplexes provides photoprotection to the PSI reaction center by channeling excitations to the IsiA protein where they are dissipated.^27−30^ However, the OCP is not found in *S. elongatus* PCC 7942, and the fate of excess excitations in this PBS complex is not as well-understood.

To uncover exciton quenching time scales in the PBS of *S. elongatus* PCC 7942 and pinpoint the sites of excess exciton quenching, we perform fluence-dependent ultrafast broadband pump–probe spectroscopy on this protein complex. This method has been used in the past to observe FRET frustration in Cy5 dyes on DNA^31^ and exciton–exciton annihilation in monolayer MoS_2_^32^ and to decode exciton equilibration time scales in LHCII^33^ trimers and LH2 membranes.^34^ We employ pump fluences corresponding to 1.3, 3.5, 6.1, and 13.6 excitations per phycocyanin rod of the phycobilisome.

We find that excess excitations created in the phycocyanin rods of the complex are annihilated before they transfer to the allophycocyanin core. This finding is unexpected in the context of previous work on LHCII and LH2 that has shown that exciton–exciton annihilation occurs primarily in the lowest-energy chromophores in these antenna complexes.^21,22^ This effect likely occurs because the core has fewer chromophores than the rods.^9−11,35−37^

Our transient differential transmission data for the different pump fluences fits well to a simple second-order annihilation model,^6,33^ and we recover exciton hopping times between bilin chromophores on adjacent stacked phycocyanin trimers from the model.^6,33^ Our recovered hopping times are in excellent agreement with earlier theoretical hopping time calculations and experiments.^16,38,39^

## Methods

### Phycobilisome Isolation

Phycobilisome complexes are isolated from wild-type *S. elongatus* PCC 7942. Cells are grown in BG-11 medium under white room lights at room temperature. The isolation is adapted from the procedure of Kirilovsky and co-workers.^40^ Cells are pelleted for 40 min at 4000 rpm. The pelleted cells are resuspended in 1 M potassium phosphate buffer and washed at 4000 rpm for 40 min multiple times to remove the BG-11 medium before the final resuspension in the 1 M phosphate buffer. Visible impurities are removed after every wash. Resuspended cells are vortex mixed with glass beads in a 1:1 volume ratio of beads and suspension three times for 1 min. To avoid local heating, the suspensions are placed on ice for 1 min after every vortex cycle. The lysed cells are incubated for 30 min with Triton X-100 (2% v/v) at 28 °C in the dark. The phycobilisome supernatant is separated from cell and glass debris by centrifugation at 20 000 rpm for 20 min. This procedure is repeated several times to maximize the phycobilisome yield. The recovered supernatant is loaded on a discontinuous sucrose gradient of 1.5, 0.75, 0.5, and 0.25 M sucrose in 1 M potassium phosphate buffer (Figure 1a). The gradients are ultracentrifuged overnight at 24 000 rpm. The deep-blue 1.5 M sucrose band is isolated and stored in −71 °C for subsequent spectroscopic measurements. Phycobilisome integrity is confirmed with gel chromatography (Figure S1 and Table S1 in the Supporting Information), fluorescence spectroscopy, and circular dichroism (CD) spectroscopy (Figure S2 in the Supporting Information). Our gel shows a prominent PC band at ∼20 kDa, confirming that the PC complexes make it to the heaviest sucrose fraction as intact phycobilisomes.^41^

### Absorption, Circular Dichroism Spectroscopy, and Fluorescence Spectroscopy

The absorption spectrum of the phycobilisome is obtained in a 1 mm path length cuvette in an Agilent Cary 5000 spectrometer. Two-dimensional fluorescence spectra are obtained in a 1 cm cuvette with 0.05 OD sample in a Horiba Jobin Yvon Fluorolog-3 spectrophotometer. CD spectra are obtained in a 1 mm cuvette on a 1.0 OD sample in a Jasco J-1500 CD spectrometer.

### Ultrafast Broadband Pump–Probe Spectroscopy

Sub-40-fs pulses centered at ∼800 nm with an average power of 2.7 W and a repetition rate of 5 kHz are generated in a Ti:sapphire Coherent Legend Elite regenerative amplifier seeded by a Coherent Micra Ti:sapphire oscillator. The laser beam is focused in argon gas at 18 psi. A portion of the resulting white-light supercontinuum is compressed to ∼10 fs using an SLM-based pulse shaper (Biophotonic Solutions Inc. MIIPSBOX640). A representative laser spectrum is shown in Figure 1c. The compressed pulse is split into pump and probe beams with a 90/10 beam splitter. The pump beam is passed through a mechanical delay stage (Aerotech) and chopped at 2.5 kHz (Newport Corp.). The pump and probe are focused into a 200 μm sample cuvette, and the beam size is characterized to be ∼290 μm. The pump and probe polarization are kept identical. The probe is then aligned with a Shamrock spectrometer and resolved at a Teledyne Dalsa Spyder 3 CCD camera. The pump energies are attenuated to 14, 35, 61, and 146 nJ per pulse using neutral density filters for the annihilation measurements, and the probe intensity is attenuated by 2 orders of magnitude.

## Results and Discussion

**Figure 2:**
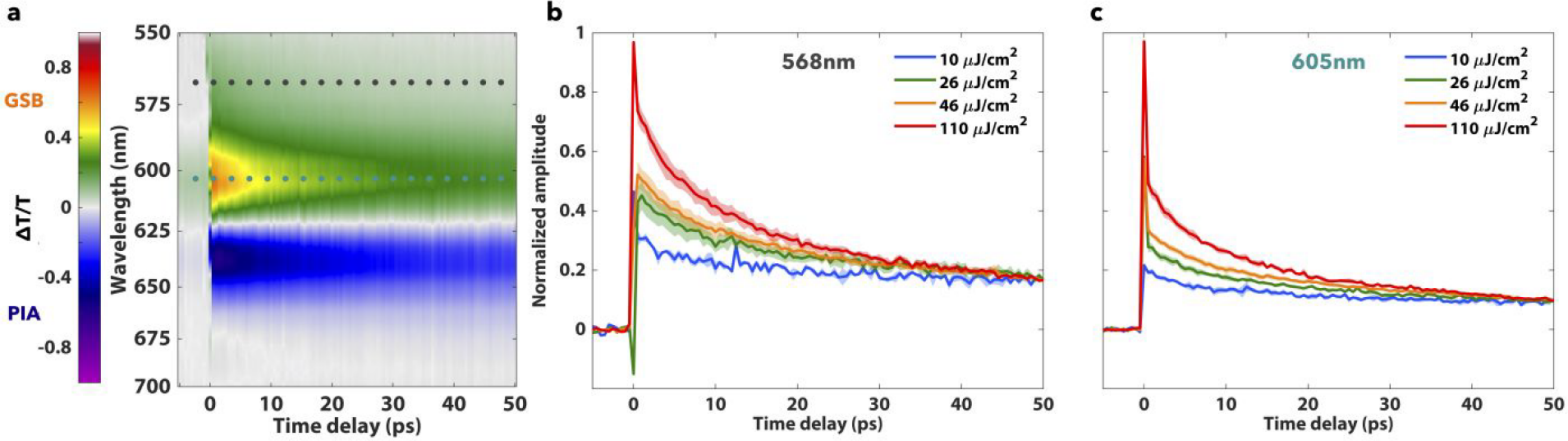
(a) Averaged transient differential transmission map for the 46 μJ/cm^2^ pump fluence. The ground-state bleach is positive, and the photoinduced absorption is negative. (b, c) Differential transmission as a function of the pump–probe delay at 568 and 605 nm for 10, 26, 46, and 110 μJ/cm^2^ pump fluences normalized to 50 ps. Error bars show the standard error.

Transient differential transmission data for isolated PBS complexes is shown in Figure 2. Two main spectroscopic features are observed in the transient transmission data (Figure 2a) for all fluences: a decaying positive feature between 560 and 620 nm, peaking at 605 nm, and a decaying negative feature red of 625 nm, peaking at 632 nm. Here, positive features correspond to the ground-state bleach, and stimulated emission signals and negative features correspond to the photoinduced absorption signal. A strong fluence dependence of the differential transmission signal decay is seen throughout the positive feature on the 50 ps time scale (Figure 2b,c) across the four fluences. A weaker fluence dependence is seen in the negative feature and cannot be deconvolved from the standard error of our measurements. The weak fluence dependence is likely seen due to spectral congestion among APC, PC bleach, and electrochromic signals in this wavelength region.

Pump–probe and time-resolved fluorescence studies have uncovered exciton dynamics in phycobilisome complexes of many cyanobacterial species. Earlier transient absorption studies also show a prominent ground-state bleach and photoinduced absorption signals in the phycobilisomes of *T. vulcanus*,^42^*Synechocystis* sp. PCC 6803,^11^ and *A. platensis*(43) in the same spectral region. The positive feature seen in our data has been previously attributed to the ground-state bleaching of the PC and APC phycocyanobilin chromophores.^43,44^ Previous studies suggest that the red negative signature observed in the transient transmission plot is not caused by the excited-state absorption of excited phycobilin molecules but is due to an electrochromic shift of phycobilin chromophores that are neighbors of excited chromophores.^43,44^ We see the same fluence dependence trend in this feature (Figure S4 in the Supporting Information), but we do not analyze it further due to the significant spectral congestion in this region and the measurements falling within the error of our experiments.

Largely overlapping absorption spectra of phycocyanin and allophycocyanin chromophores lead to spectral congestion and confound the deconvolution of allophycocyanin specific dynamics in this PBS complex.^45,46^ The WT *S. elongatus* phycobilisome contains 324 phycocyanin chromophores as opposed to 48 allophycocyanin chromophores, thus adding to the difficulty of separating the core-specific signal or rod to core-transfer dynamics. Mutants with truncated rods or no rods have been designed to study these dynamics specifically.^11^ However, to selectively observe exciton–exciton annihilation and decay dynamics in the phycocyanin rods, we look at the blue side of the feature near 568 nm where allophycocyanin absorption is minimal:^43^ a previous pump–probe study on *A. variabilis* allophycocyanin trimers showed an undetectable ground-state bleach signal blue of 620 nm.^47^ Fluence-dependent transient differential transmission plots at 568 nm for the different light fluences used in our experiment are shown in Figure 2b. The dynamics show a clear fluence dependence, with higher fluences leading to faster signal decays. This fluence dependence is also seen through the entire positive feature, but we restrict our discussion to the bluest signal region to prevent the convolution of PC rod dynamics with APC core dynamics which are also excited in our broadband experiments. A biexponential fit (Figure S4 in the Supporting Information) for these curves yields a 7–10 ps component (Table S3 in the Supporting Information) that remains relatively unchanged with fluence and a second time constant that decreases from ∼150 to ∼50 ps as the fluence increases. Previous studies have attributed a 150–200 ps time constant to exciton transfer from PC to APC,^43^ and the sub-10-ps time constant can be attributed to intrahexamer exciton relaxation within phycocyanins from the work of Kirilovsky and co-workers.^24^ The changing longer time constant suggests that multiexciton events occur as the fluence increases in the PC rods of this PBS.

**Figure 3:**
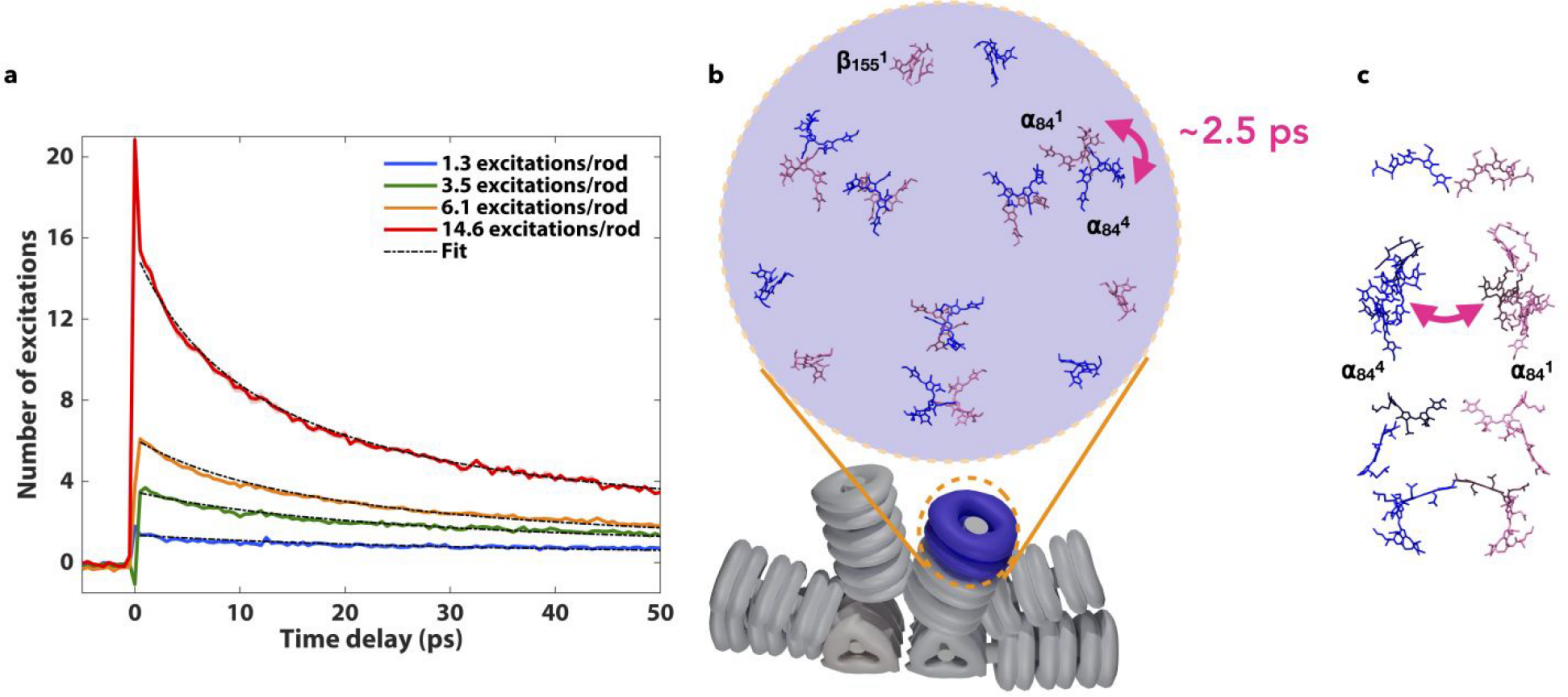
(a) Second-order fits for the transient differential transmission signal at 568 nm for the different number of excitations per rod calculated for our fluences. (b) Recovered hopping times of ∼2.5 ps correspond to exciton hops between α_84_^1^ and α_84_^1^ (shown in pink and blue, respectively) chromophores of adjacent monomers within a PC trimer. (c) Side view of the two stacked trimers.

To determine if the fluence dependence arises from exciton–exciton annihilation, we first calculate the number of excitations on each rod for the four different light fluences.^31,45,48^ We obtain values of 1.3, 3.1, 6.5, and 14.6 initial excitations per rod, defined as *N*(0), for the 10, 26, 46, and 110 μJ/cm^2^ fluences, respectively. The calculation of the number of excitations is detailed in the Supporting Information. We model the exciton–exciton annihilation process after a simple second-order differential equation^32^
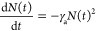
1where *N*(*t*) is the number of excitations per rod at a given time *t* and γ_a_ is the rate constant for exciton–exciton annihilation. Solving for *N*(*t*) gives
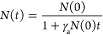
2We fit the obtained transient differential transmission data for all fluences to this equation and the calculated *N*(0) values to this equation. The fits of our data and the calculated initial number of excitations are shown in Figure 3a. The annihilation rate is obtained through our fits for the different fluences. The annihilation rates are reported in Table S2 in the Supporting Information. A wide range of FRET hopping rates (500 fs to 50 ps) have been calculated for different chromophore pairs in the PC rods,^16^ and annihilation likely occurs through a combination of these hops with a large contribution from annihilation of between α_84_ and β_84_ chromophores on adjacent trimers in addition to the closest α_84_ and β_155_ within a trimer.

Using the obtained exciton–exciton annihilation rate, we calculate the mean exciton hopping time for the four fluences using the formula^6,33^
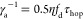
3where η is the total number of chromophores involved in the hopping process (54 per rod), *f*_d_ is the fractal dimension of the lattice on which exciton hopping occurs, and *τ*_hop_ is the unknown parameter, which is the mean exciton hopping time. The chromophore lattice of phycocyanin rods is not ordered, and its fractal dimension has not been reported to our knowledge. Therefore, we assume *f*_d_ to be 1 for the phycocyanin rods because this value has not been calculated but ranges from 0.7 to 1 for most photosynthetic complexes.^6,32−34^ We note that using *f*_d_ = 0.7 will still yield good agreement with the calculated FRET hopping rate between the α_84_ and β_84_ chromophores on adjacent trimers (Figure 3c). We obtain a *τ*_hop_ value range of 2.2–6.4 ps using eq 3. The hopping time obtained through our annihilation measurements is likely a weighted average of FRET hopping times between different chromophore pairs that have been previously calculated and reported. However, it matches calculated FRET hopping times between α_84_^1^ and α_84_^4^ chromophores and β_155_^1^ and β_155_^6^ chromophores on adjacent stacked trimers.^16^ It is likely that the calculated hopping time increases with fluence due to a higher probability of simultaneously exciting the β_155_^1^ and α_84_^1^ chromophores between which the hopping is slower.^16^ Previous studies have shown that transfer between the most closely spaced α_84_^1^ and β_84_^1^ occurs on the subpicosecond time scale.^38,42,46,49^ The predicted longer hopping times^16^ between the farther apart α_84_^1^ and β_155_^1^ have also been experimentally observed before.^37,49−53^ A recent time-resolved fluorescence study observed a 4 nm FRET distance and a 90% transfer quantum yield between nearest chromophores of PC and photosystem I in rod-only PBS complexes.^54^ Here, we experimentally determine the hopping time along a phycobilisome rod between isoenergetic α_84_ and β_84_ chromophores of adjacent PC trimers using our annihilation measurements. Our fits coupled with the ∼140 ps time constant of PC to APC hops confirm that the difference in fluence-dependent dynamics at 568 nm is seen due to exciton–exciton annihilation within PC rods and before transfer to APC cores.

In summary, we have shown that excess excitons created in the highest-energy phycobilin chromophores in the phycobilisome complex of cyanobacteria *S. elongatus* PCC 7942 can quench through an exciton–exciton annihilation mechanism within the PC rods before they hop downhill to the APC. While a single rod would receive two excitations together approximately only once in 2 days when the peak solar intensity is 1000 W/m^2^, fast annihilation within phycocyanin rods could prevent the formation of triplet excitons in the photosystems which are also sites of multiple exciton generation in high fluences. Our observations could explain in part the fluorescence quenching observed in an earlier single-molecule fluorescence study on the *Synechocystis* phycobilisome that was excited monochromatically at 590 nm.^26^ While our study uses higher than physiological fluences^26,55,56^ to elucidate exciton–exciton annihilation in phycocyanin rods, this mechanism could operate a few times in every rod in a phycobilisome complex over a cell’s lifetime. This finding is especially relevant in the scenario when other rapid internal conversion pathways of exciton decay in the phycobilisome, including OCP binding , are absent. A previous study showed intra- and interdisk annihilation in the allophycocyanin core of the *Synechocystis* phycobilisome but did not explore annihilation in the C-phycocyanin rods.^11^ Annihilation in C-phycocyanin trimers has been shown to be far less efficient than in allophycocyanin in previous studies,^37,57^ and our obtained biexponential fits show a much faster ∼10 ps time constant that is absent in these trimers, suggesting that the formation of hexamers opens faster decay and transfer channels. Our calculations also show that this effect would occur in individual phycobilisome rods several times over a cellular lifetime. In conjunction with the comparison to other antenna complexes, we show that the annihilation seen in phycocyanin rods is physiologically relevant, an emergent property of the complex assembly and different from annihilation seen in LHCII in which the fluence dependence is seen only in the reddest wavelength region.^22^

Finally, our findings experimentally recover FRET-predicted exciton hopping times of 2.2–6.5 ps between chromophores on adjacent PC trimers in the PBS rods. The obtained time constant complements previous seminal fluorescence and time-resolved anisotropy studies that aim to make a structural model of the cyanobacterial light-harvesting antenna.^58^

## Supporting Information Available

Additional transient transmission spectra, protein characterization, data fitting, and a calculation of the number of excitations (PDF)

## References

[ref1] Kasting J. F. Life and the Evolution of Earth’s Atmosphere. Science 2002, 296 (5570), 1066–1068. 10.1126/science.1071184. 12004117

[ref2] Green B.; Parson W. W. Light-Harvesting Antennas in Photosynthesis; Advances in Photosynthesis and Respiration; Springer: The Netherlands, 2003.

[ref3] Blankenship R. Molecular Mechanisms of Photosynthesis, 2nd ed.; Wiley Blackwell: Oxford, 2014.

[ref4] Scholes G. D.; Fleming G. R.; Olaya-Castro A.; van Grondelle R. Lessons from Nature about Solar Light Harvesting. Nat. Chem. 2011, 3 (10), 763–774. 10.1038/nchem.1145. 21941248

[ref5] Liu H.; Zhang H.; Niedzwiedzki D. M.; Prado M.; He G.; Gross M. L.; Blankenship R. E. Phycobilisomes Supply Excitations to Both Photosystems in a Megacomplex in Cyanobacteria. Science 2013, 342 (6162), 1104–1107. 10.1126/science.1242321. 24288334 PMC3947847

[ref6] van Amerongen H.; van Grondelle R.; Valkunas L. Photosynthetic Excitons; World Scientific, 2000.

[ref7] Homoelle B. J.; Edington M. D.; Diffey W. M.; Beck W. F. Stimulated Photon-Echo and Transient-Grating Studies of Protein-Matrix Solvation Dynamics and Interexciton-State Radiationless Decay in α Phycocyanin and Allophycocyanin. J. Phys. Chem. B 1998, 102 (16), 3044–3052. 10.1021/jp972782x.

[ref8] Homoelle B. J.; Beck W. F. Solvent Accessibility of the Phycocyanobilin Chromophore in the Alpha Subunit of C-Phycocyanin: Implications for a Molecular Mechanism for Inertial Protein-Matrix Solvation Dynamics. Biochemistry 1997, 36 (42), 12970–12975. 10.1021/bi971121n. 9335557

[ref9] Marx A.; Adir N. Allophycocyanin and Phycocyanin Crystal Structures Reveal Facets of Phycobilisome Assembly. Biochim. Biophys. Acta BBA - Bioenerg. 2013, 1827 (3), 311–318. 10.1016/j.bbabio.2012.11.006. 23201474

[ref10] Calzadilla P. I.; Muzzopappa F.; Sétif P.; Kirilovsky D. Different Roles for ApcD and ApcF in *Synechococcus elongatus* and *Synechocystis sp.* PCC 6803 Phycobilisomes. Biochim. Biophys. Acta BBA - Bioenerg. 2019, 1860 (6), 488–498. 10.1016/j.bbabio.2019.04.004. 31029593

[ref11] van Stokkum I. H. M.; Gwizdala M.; Tian L.; Snellenburg J. J.; van Grondelle R.; van Amerongen H.; Berera R. A Functional Compartmental Model of the *Synechocystis* PCC 6803 Phycobilisome. Photosynth. Res. 2018, 135 (1–3), 87–102. 10.1007/s11120-017-0424-5. 28721458 PMC5784004

[ref12] Womick J. M.; Moran A. M. Vibronic Enhancement of Exciton Sizes and Energy Transport in Photosynthetic Complexes. J. Phys. Chem. B 2011, 115 (6), 1347–1356. 10.1021/jp106713q. 21268650

[ref13] Womick J. M.; Moran A. M. Exciton Coherence and Energy Transport in the Light-Harvesting Dimers of Allophycocyanin. J. Phys. Chem. B 2009, 113 (48), 15747–15759. 10.1021/jp907644h. 19894754

[ref14] Womick J. M.; Miller S. A.; Moran A. M. Toward the Origin of Exciton Electronic Structure in Phycobiliproteins. J. Chem. Phys. 2010, 133 (2), 024507 10.1063/1.3457378. 20632763

[ref15] Ying L.; Xie X. S. Fluorescence Spectroscopy, Exciton Dynamics, and Photochemistry of Single Allophycocyanin Trimers. J. Phys. Chem. B 1998, 102 (50), 10399–10409. 10.1021/jp983227d.

[ref16] Sauer K.; Scheer H. Excitation Transfer in C-Phycocyanin. Förster Transfer Rate and Exciton Calculations Based on New Crystal Structure Data for C-Phycocyanins from *Agmenellum quadruplicatum* and *Mastigocladus laminosus*. Biochim. Biophys. Acta BBA -Bioenerg 1988, 936 (2), 157–170. 10.1016/0005-2728(88)90232-0.

[ref17] Hays A.-M. A.; Vassiliev I. R.; Golbeck J. H.; Debus R. J. Role of D1-His190 in the Proton-Coupled Oxidation of Tyrosine YZ in Manganese-Depleted Photosystem II. Biochemistry. 1999, 38 (37), 11851–11865. 10.1021/bi990716a. 10508388

[ref18] Schenck H. On the Focusing of Sunlight by Ocean Waves. J. Opt. Soc. Am. 1957, 47 (7), 653–657. 10.1364/JOSA.47.000653.

[ref19] Darecki M.; Stramski D.; Sokólski M. Measurements of High-Frequency Light Fluctuations Induced by Sea Surface Waves with an Underwater Porcupine Radiometer System. J. Geophys. Res. 2011, 116, C00H09 10.1029/2011JC007338.

[ref20] Lee Y.; Gorka M.; Golbeck J. H.; Anna J. M. Ultrafast Energy Transfer Involving the Red Chlorophylls of Cyanobacterial Photosystem I Probed through Two-Dimensional Electronic Spectroscopy. J. Am. Chem. Soc. 2018, 140 (37), 11631–11638. 10.1021/jacs.8b04593. 30133281

[ref21] Bruggemann B.; May V. Exciton Exciton Annihilation Dynamics in Chromophore Complexes. II. Intensity Dependent Transient Absorption of the LH2 Antenna System. J. Chem. Phys. 2004, 120 (5), 2325–2336. 10.1063/1.1637585. 15268371

[ref22] Bittner T.; Irrgang K.-D.; Renger G.; Wasielewski M. R. Ultrafast Excitation Energy Transfer and Exciton-Exciton Annihilation Processes in Isolated Light Harvesting Complexes of Photosystem II (LHC II) from Spinach. J. Phys. Chem. 1994, 98 (46), 11821–11826. 10.1021/j100097a004.

[ref23] Squires A. H.; Dahlberg P. D.; Liu H.; Magdaong N. C. M.; Blankenship R. E.; Moerner W. E. Single-Molecule Trapping and Spectroscopy Reveals Photophysical Heterogeneity of Phycobilisomes Quenched by Orange Carotenoid Protein. Nat. Commun. 2019, 10 (1), 1172 10.1038/s41467-019-09084-2. 30862823 PMC6414729

[ref24] Berera R.; van Stokkum I. H. M.; Gwizdala M.; Wilson A.; Kirilovsky D.; van Grondelle R. The Photophysics of the Orange Carotenoid Protein, a Light-Powered Molecular Switch. J. Phys. Chem. B 2012, 116 (8), 2568–2574. 10.1021/jp2108329. 22257008

[ref25] Kirilovsky D.; Kerfeld C. A. Cyanobacterial Photoprotection by the Orange Carotenoid Protein. Nat. Plants. 2016, 2 (12), 16180 10.1038/nplants.2016.180. 27909300

[ref26] Gwizdala M.; Berera R.; Kirilovsky D.; van Grondelle R.; Krüger T. P. J. Controlling Light Harvesting with Light. J. Am. Chem. Soc. 2016, 138 (36), 11616–11622. 10.1021/jacs.6b04811. 27546794

[ref27] Chen H.-Y. S.; Niedzwiedzki D. M.; Bandyopadhyay A.; Biswas S.; Pakrasi H. B. A Novel Mode of Photoprotection Mediated by a Cysteine Residue in the Chlorophyll Protein IsiA. MBio 2021, 12 ( (1), ), 10.1128/mBio.03663-20. PMC8545134 33593975

[ref28] Havaux M.; Guedeney G.; Hagemann M.; Yeremenko N.; Matthijs H. C. P.; Jeanjean R. The Chlorophyll-Binding Protein IsiA Is Inducible by High Light and Protects the Cyanobacterium *Synechocystis* PCC6803 from Photooxidative Stress. FEBS Lett. 2005, 579 (11), 2289–2293. 10.1016/j.febslet.2005.03.021. 15848160

[ref29] van der Weij-de Wit C. D.; Ihalainen J. A.; van de Vijver E.; D’Haene S.; Matthijs H. C. P.; van Grondelle R.; Dekker J. P. Fluorescence Quenching of IsiA in Early Stage of Iron Deficiency and at Cryogenic Temperatures. Biochim. Biophys. Acta 2007, 1767 (12), 1393–1400. 10.1016/j.bbabio.2007.10.001. 17980697

[ref30] Andrizhiyevskaya E. G.; Schwabe T. M. E.; Germano M.; D’Haene S.; Kruip J.; van Grondelle R.; Dekker J. P. Spectroscopic Properties of PSI-IsiA Supercomplexes from the Cyanobacterium *Synechococcus* PCC 7942. Biochim. Biophys. Acta 2002, 1556 (2–3), 265–272. 10.1016/S0005-2728(02)00371-7. 12460685

[ref31] Mazuski R. J.; Díaz S. A.; Wood R. E.; Lloyd L. T.; Klein W. P.; Mathur D.; Melinger J. S.; Engel G. S.; Medintz I. L. Ultrafast Excitation Transfer in Cy5 DNA Photonic Wires Displays Dye Conjugation and Excitation Energy Dependency. J. Phys. Chem. Lett. 2020, 11 (10), 4163–4172. 10.1021/acs.jpclett.0c01020. 32391695

[ref32] Sun D.; Rao Y.; Reider G. A.; Chen G.; You Y.; Brézin L.; Harutyunyan A. R.; Heinz T. F. Observation of Rapid Exciton–Exciton Annihilation in Monolayer Molybdenum Disulfide. Nano Lett. 2014, 14 (10), 5625–5629. 10.1021/nl5021975. 25171389

[ref33] Barzda V.; Gulbinas V.; Kananavicius R.; Cervinskas V.; van Amerongen H.; van Grondelle R.; Valkunas L. Singlet–Singlet Annihilation Kinetics in Aggregates and Trimers of LHCII. Biophys. J. 2001, 80 (5), 2409–2421. 10.1016/S0006-3495(01)76210-8. 11325740 PMC1301429

[ref34] Dahlberg P. D.; Ting P.-C.; Massey S. C.; Allodi M. A.; Martin E. C.; Hunter C. N.; Engel G. S. Mapping the Ultrafast Flow of Harvested Solar Energy in Living Photosynthetic Cells. Nat. Commun. 2017, 8 (1), 988 10.1038/s41467-017-01124-z. 29042567 PMC5715167

[ref35] Holzwarth A. R. Structure-Function Relationships and Energy Transfer in Phycobiliprotein Antennae. Physiol. Plant. 1991, 83 (3), 518–528. 10.1034/j.1399-3054.1991.830328.x.

[ref36] Adir N.; Bar-Zvi S.; Harris D. The Amazing Phycobilisome. Light Harvest. 2020, 1861 (4), 148047 10.1016/j.bbabio.2019.07.002. 31306623

[ref37] van Grondelle R. Excitation Energy Transfer, Trapping and Annihilation in Photosynthetic Systems. Biochim. biophys. acta. 1985, 811 (2), 147–195. 10.1016/0304-4173(85)90017-5.

[ref38] Gillbro T.; Sharkov A. V.; Kryukov I. V.; Khoroshilov E. V.; Kryukov P. G.; Fischer R.; Scheer H. Förster Energy Transfer between Neighbouring Chromophores in C-Phycocyanin Trimers. Biochim. Biophys. Acta Bioenerg. 1993, 1140 (3), 321–326. 10.1016/0005-2728(93)90072-N.

[ref39] Womick J. M.; Moran A. M. Nature of Excited States and Relaxation Mechanisms in C-Phycocyanin. J. Phys. Chem. B 2009, 113 (48), 15771–15782. 10.1021/jp908093x. 19902910

[ref40] Gwizdala M.; Wilson A.; Kirilovsky D. In Vitro Reconstitution of the Cyanobacterial Photoprotective Mechanism Mediated by the Orange Carotenoid Protein in *Synechocystis* PCC 6803. The Plant Cell. 2011, 23 (7), 2631–2643. 10.1105/tpc.111.086884. 21764991 PMC3226224

[ref41] Sato T.; Minagawa S.; Kojima E.; Okamoto N.; Nakamoto H. HtpG, the Prokaryotic Homologue of Hsp90, Stabilizes a Phycobilisome Protein in the Cyanobacterium *Synechococcus elongatus* PCC 7942: Interaction of HtpG with an in Vivo Substrate. Mol. Microbiol. 2010, 76 (3), 576–589. 10.1111/j.1365-2958.2010.07139.x. 20345653

[ref42] Nganou C.; David L.; Adir N.; Mkandawire M. Linker Proteins Enable Ultrafast Excitation Energy Transfer in the Phycobilisome Antenna System of *Thermosynechococcus vulcanus*. Photochem. Photobiol. Sci. 2016, 15 (1), 31–44. 10.1039/C5PP00285K. 26537632

[ref43] Fălămaş A.; Porav S. A.; Tosa V. Investigations of the Energy Transfer in the Phycobilisome Antenna of *Arthrospira platensis* Using Femtosecond Spectroscopy. Appl. Sci. 2020, 10 (11), 4045 10.3390/app10114045.

[ref44] Niedzwiedzki D. M.; Bar-Zvi S.; Blankenship R. E.; Adir N. Mapping the Excitation Energy Migration Pathways in Phycobilisomes from the Cyanobacterium *Acaryochloris marina*. Biochim. Biophys. Acta BBA - Bioenerg. 2019, 1860 (4), 286–296. 10.1016/j.bbabio.2019.01.002. 30703363

[ref45] Grabowski J.; Gantt E. Photophysical Properties of Phycobiliproteins from Phycobilisomes: Fluorescence Lifetimes, Quantum Yields, and Polarization Spectra. Photochem. Photobiol. 1978, 28 (1), 39–45. 10.1111/j.1751-1097.1978.tb06927.x.

[ref46] Glazer A. N.; Fang S.; Brown D. M. Spectroscopic Properties of C-Phycocyanin and of Its α and β Subunits. J. Biol. Chem. 1973, 248 (16), 5679–5685. 10.1016/S0021-9258(19)43559-X. 4198883

[ref47] Shiu Y. J.; Zhang J. M.; Hayashi M.; Gulbinas V.; Yang C. M.; Lin S. H. A Transient Absorption Study of Allophycocyanin. J. Chem. Sci. 2002, 114 (6), 611–621. 10.1007/BF02708855.

[ref48] Dostál J.; Fennel F.; Koch F.; Herbst S.; Würthner F.; Brixner T. Direct Observation of Exciton–Exciton Interactions. Nat. Commun. 2018, 9 (1), 2466 10.1038/s41467-018-04884-4. 29941915 PMC6018121

[ref49] Riter R. E.; Edington M. D.; Beck W. F. Isolated-Chromophore and Exciton-State Photophysics in C-Phycocyanin Trimers. J. Phys. Chem. B 1997, 101 (13), 2366–2371. 10.1021/jp962609l.

[ref50] Xia A.; Zhu J.; Wu H.; Jiang L.; Zhang X.; Sudha M.; MaruthiSai P. S. Time-Resolved Polarized Absorption of C-Phycocyanin from the Cyanobacterium *Westiellopsisprolifica*. J. Photochem. Photobiol., B 1993, 19 (2), 111–117. 10.1016/1011-1344(93)87104-U.

[ref51] Debreczeny M. P.; Sauer K.; Zhou J.; Bryant D. A. Comparison of Calculated and Experimentally Resolved Rate Constants for Excitation Energy Transfer in C-Phycocyanin. 2. Trimers. J. Phys. Chem. 1995, 99 (20), 8420–8431. 10.1021/j100020a081.

[ref52] Debreczeny M. P.; Sauer K.; Zhou J.; Bryant D. A. Comparison of Calculated and Experimentally Resolved Rate Constants for Excitation Energy Transfer in C-Phycocyanin. 1. Monomers. J. Phys. Chem. 1995, 99 (20), 8412–8419. 10.1021/j100020a080.

[ref53] Xia A. D.; Zhu J. C.; Jiang L. J.; Li D. L.; Zhang X. Y. Energy Transfer Kinetics in C-Phycocyanin from Cyanobacterium *Wastiellopsisprolifica* Studied by Pump-Probe Techniques. Biochem. Biophys. Res. Commun. 1991, 179 (1), 558–564. 10.1016/0006-291X(91)91407-4. 1909122

[ref54] Noji T.; Watanabe M.; Dewa T.; Itoh S.; Ikeuchi M. Direct Energy Transfer from Allophycocyanin-Free Rod-Type CpcL-Phycobilisome to Photosystem I. J. Phys. Chem. Lett. 2021, 12, 6692–6697. 10.1021/acs.jpclett.1c01763. 34260249

[ref55] Krüger T. P. J.; Ilioaia C.; Valkunas L.; van Grondelle R. Fluorescence Intermittency from the Main Plant Light-Harvesting Complex: Sensitivity to the Local Environment. J. Phys. Chem. B 2011, 115 (18), 5083–5095. 10.1021/jp109833x. 21452801

[ref56] Krüger T. P. J.; Ilioaia C.; Johnson M. P.; Ruban A. V.; Papagiannakis E.; Horton P.; van Grondelle R. Controlled Disorder in Plant Light-Harvesting Complex II Explains Its Photoprotective Role. Biophys. J. 2012, 102 (11), 2669–2676. 10.1016/j.bpj.2012.04.044. 22713583 PMC3368130

[ref57] Doukas A. G.; Stefancic V.; Buchert J.; Alfano R.; Zilinskas B. A. Exciton Annihilation in the Isolated Phycobiliproteins from the Blue-Green Alga *Nostoc* Sp. Using Picosecond Absorption Spectroscopy. Photochem. Photobiol. 1981, 34 (4), 505–510. 10.1111/j.1751-1097.1981.tb09393.x.

[ref58] Zhang J.-M.; Zhao J.-Q.; Jiang L.-J.; Zheng X.-G.; Zhao F.-L.; Wang H.-Z. Studies on the Energy Transfer among the Rod-Core Complex from Phycobilisome of *Anabaena Variabilis* by Time Resolved Fluorescence Emission and Anisotropy Spectra. Biochim. Biophys. Acta Bioenerg. 1997, 1320 (3), 285–296. 10.1016/S0005-2728(97)00032-7.

