# Supplementary material for: Annihilation of Excess Excitations along Phycocyanin Rods Precedes Downhill Flow to Allophycocyanin Cores in the Phycobilisome of Synechococcus elongatus PCC 7942
Source: J Phys Chem B. 2022 Jan 4;126(1):23–9. doi: 10.1021/acs.jpcb.1c06509 (PMC8762654; doi:10.1021/acs.jpcb.1c06509)
Supplement: Supplementary file 1 — jp1c06509_si_001.pdf [file jp1c06509_si_001.pdf]

Supporting information

Annihilation of Excess Excitations along Phycocyanin Rods Precedes Downhill Flow to Allophycocyanin Cores in the Phycobilisome of *Synechococcus elongatus* PCC 7942

Polina Navotnaya[1,△], Siddhartha Sohoni[1,△], Lawson T. Lloyd[1], Sami M. Abdulhadi[1], Po-Chieh Ting[1], Jacob S. Higgins[1], and Gregory S. Engel[1,*]

[1]Department of Chemistry, James Franck Institute and Institute for Biophysical Dynamics, The University of Chicago, Illinois 60637

[△]contributed equally

*correspondence to gsengel@uchicago.edu

Table of contents

PBS Integrity Confirmation S2

Number of Excitations in Pump-probe Experiment S5

Annihilation Model Fits Obtained from Pump-probe Spectra S6

Biexponential Fits Obtained from Pump-probe Spectra S7

Reproducibility of Pump-probe Spectra S10

Long-time Dynamics in PBS S11

Sucrose gradient unprocessed image S12

Phycobilisome fluorescence spectrum S13

Calculation of the frequency of exciton-exciton annihilation in physiological conditions S14

References S14

PBS integrity confirmation

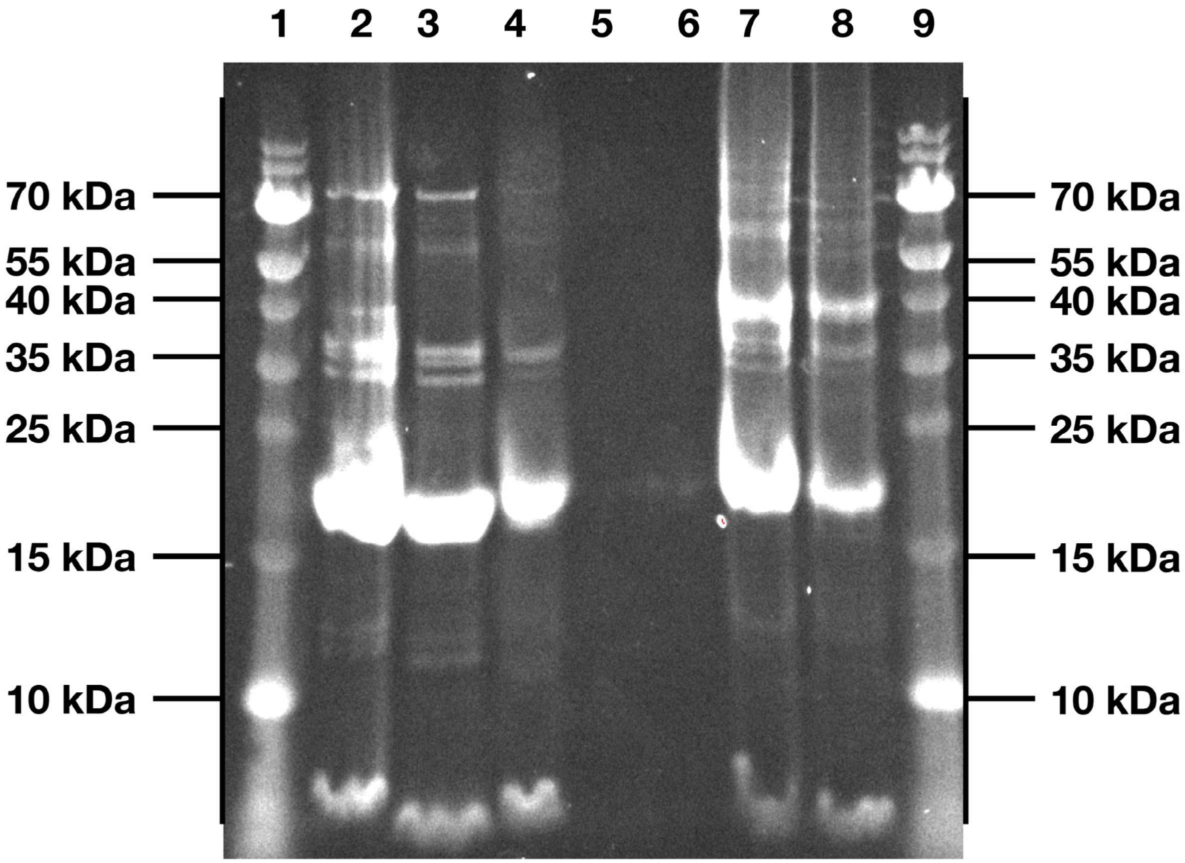

Figure S1. The 1.5 M sucrose gradient fraction with the isolated phycobilisome was tested using SDS-PAGE for integrity. Gel was confirmed with Sato, et al. (1).

Table S1: SDS-PAGE lane descriptions: Earlier protocol recommended using a non-reducing Lane Marker buffer as is shown in Lane 3:

Gel details

Lane Number	Details
1	Page Ruler – Prestained protein ladder – Thermoscientific<br>10 – 15 – 25 – 35 – 40 – 55 – 70 – 100 – 130 – 180 kDa
2	PBS + Gel Loading Buffer II (Denaturating PAGE) – 15 mins at 95 C.
3	PBS + Non-reducing Lane Marker Sample Buffer – 15 mins at 95 C
4	PBS native: not heated, no Sample Loading buffer
5	Blank
6	Blank
7	PBS + Non-reducing Lane Marker Sample Buffer – 15 mins at 95 C + Reducing Agent 2-Mercaptomethanol
8	PBS + Gel Loading Buffer II (Denaturating PAGE) – 15 mins at 95 C + Reducing Agent 2-Mercaptomethanol
9	Page Ruler – Prestained protein ladder – Thermoscientific<br>10 – 15 – 25 – 35 – 40 – 55 – 70 – 100 – 130 – 180 kDa

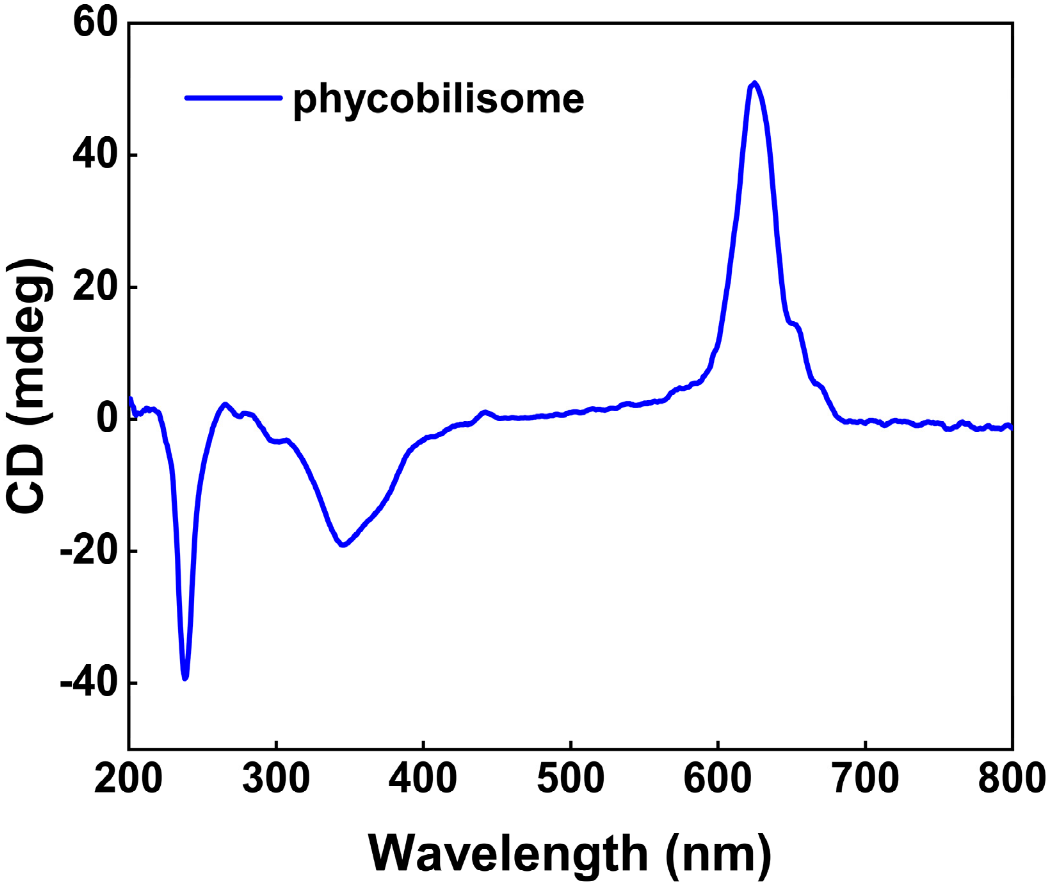

Figure S2: Circular dichroism spectrum of phycobilisome from cyanobacteria *S. elongatus* PCC 7942

Number of excitations in pump-probe experiment

To identify the number of excitons generated upon excitation, we perform the calculation adapted from Dostal and co-authors (3). The following equation describes the number of excitations per phycocyanin rod:

$$\frac{exc}{rod} = \frac{E_{pump}}{A_{overlap}} * \frac{\int Pump(\lambda) * \lambda * \left(1 - 10^{-A(\lambda)}\right) d\lambda}{hc \int Pump(\lambda) d\lambda} * \frac{1}{CN_A d}$$

Here, E_{pump} is the average energy of the pump pulse collected with Coherent LabMax-TOP powermeter, $A_{overlap}$ is the effective area of the pump and probe overlap, $Pump(\lambda)$ is the spectrum of the excitation pulse collected with Ocean Optics USB4000 Spectrometer, $A(\lambda)$ is the phycobilisome absorption spectrum shown in **Figure 1** of Main Text, C is the molar concentration of the phycocyanin rods in the solution, N_A is the Avogadro's number and *d* is the thickness of the sample cell used in pump-probe measurements. We use the following values for our calculation:

d = 200μm

ε (extinction coefficient) of one phycocyanin rod at 568nm = $3.02 * 10^6$ M^{-1} cm^{-1}

Source: Interchim Inc.

https://www.interchim.fr/ft/2/28310A.pdf?fbclid=IwAR2FJAWJno0vohgVHq7dEkVDsfzFizzq1TyW6zl7-SQwezySdgQepHKZQE8

This value is consistent with the work of Brown and co-workers for the absorption maximum at 620nm (Glazer, A. N.; Fang, S.; Brown, D. M. Spectroscopic Properties of C-Phycocyanin and of Its α and β Subunits. *J. Biol. Chem.* **1973**, *248* (16), 5679–5685.)

Concentration of our sample is calculated from this ε for an OD of 0.0689 at 568nm in 200μm and comes to $6.86 * 10^{20}$ rods/m^3.

Our overlap volume is $1.32*10^{-11}$ m^3 giving a concentration of $9.06 * 10^9$ rods/excitation volume.

Annihilation model fittings for multiple wavelengths in GSB region

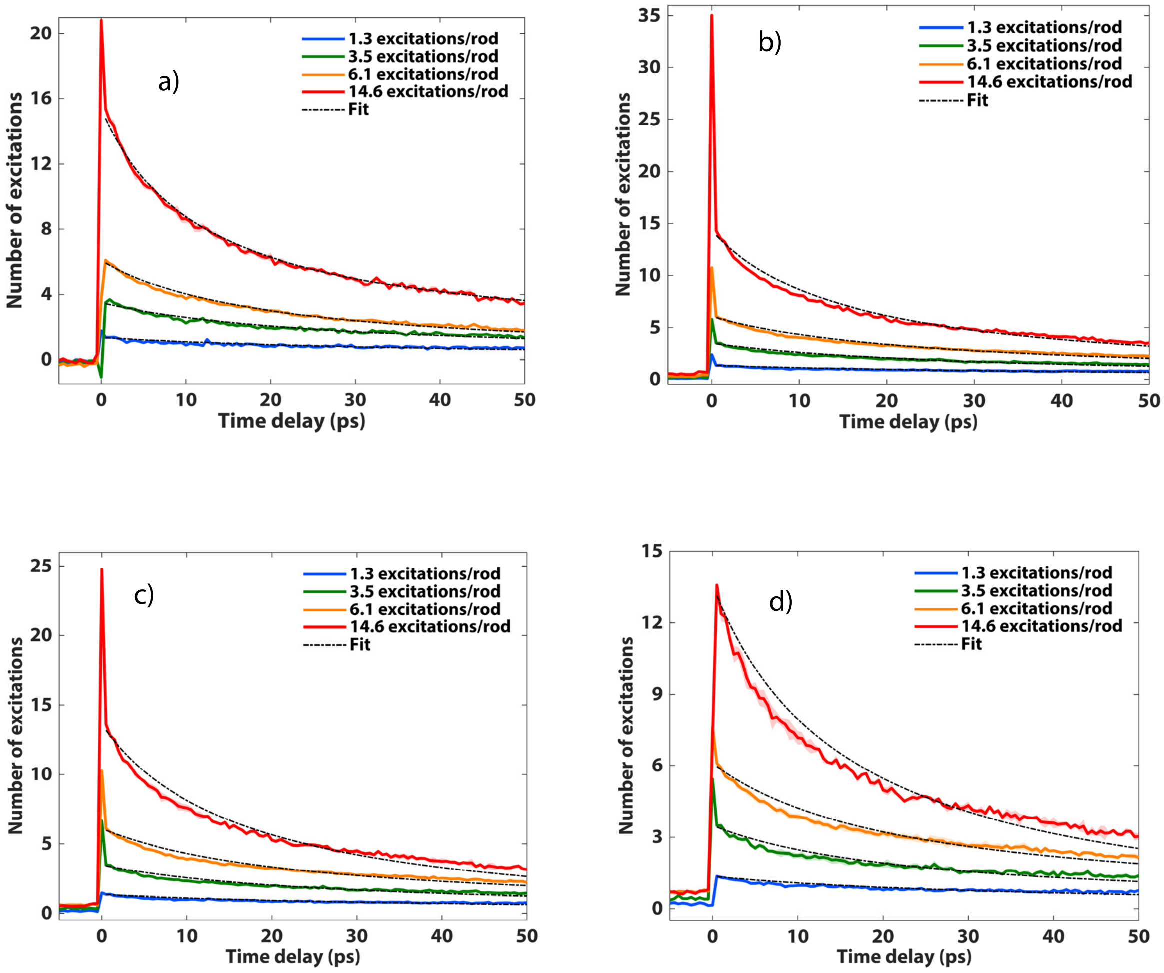

Figure S3: Fits to annihilation model at a) 568nm, b) 588nm, c) 596nm and d) 605nm. The quality of the fit decreases with the increased contribution from the allophycocyanin core.

Table S2: Annihilation rates and hopping times from the annihilation model:

		**1.3 exc/rod**	**3.5 exc/rod**	**6.1 exc/rod**	**14.6 exc/rod**
**568nm**	$\mathbf{R^2}$	0.72	0.95	0.98	0.99
	**γ (1/fs)**	$1.93x10^{-5}$	$9.78x10^{-6}$	$8.41x10^{-6}$	$5.83x10^{-6}$
	$\boldsymbol{\tau_{hop}}$ **(ps)**	2.15±0.48	3.81±0.22	4.42±0.17	6.36±0.21
**588nm**	$\mathbf{R^2}$	0.80	0.92	0.96	0.98
	**γ (1/fs)**	$1.50x10^{-5}$	$1.00x10^{-5}$	$6.74x10^{-6}$	$4.48x10^{-6}$
	$\boldsymbol{\tau_{hop}}$ **(ps)**	2.52±0.23	3.76±0.36	5.54±0.32	8.27±0.16
**596nm**	$\mathbf{R^2}$	0.75	0.91	0.94	0.96
	**γ (1/fs)**	$1.65x10^{-5}$	$1.02x10^{-5}$	$6.7x10^{-6}$	$4.04x10^{-6}$
	$\boldsymbol{\tau_{hop}}$ **(ps)**	2.25±0.10	3.64±0.28	5.57±0.31	9.20±0.37
**605nm**	$\mathbf{R^2}$	0.75	0.90	0.94	0.95
	**γ (1/fs)**	$1.93x10^{-5}$	$1.20x10^{-5}$	$7.38x10^{-6}$	$4.40x10^{-6}$
	$\boldsymbol{\tau_{hop}}$ **(ps)**	1.96±0.21	3.17±0.36	5.10±0.38	8.44±0.35

Pump-probe biexponential fitting constants

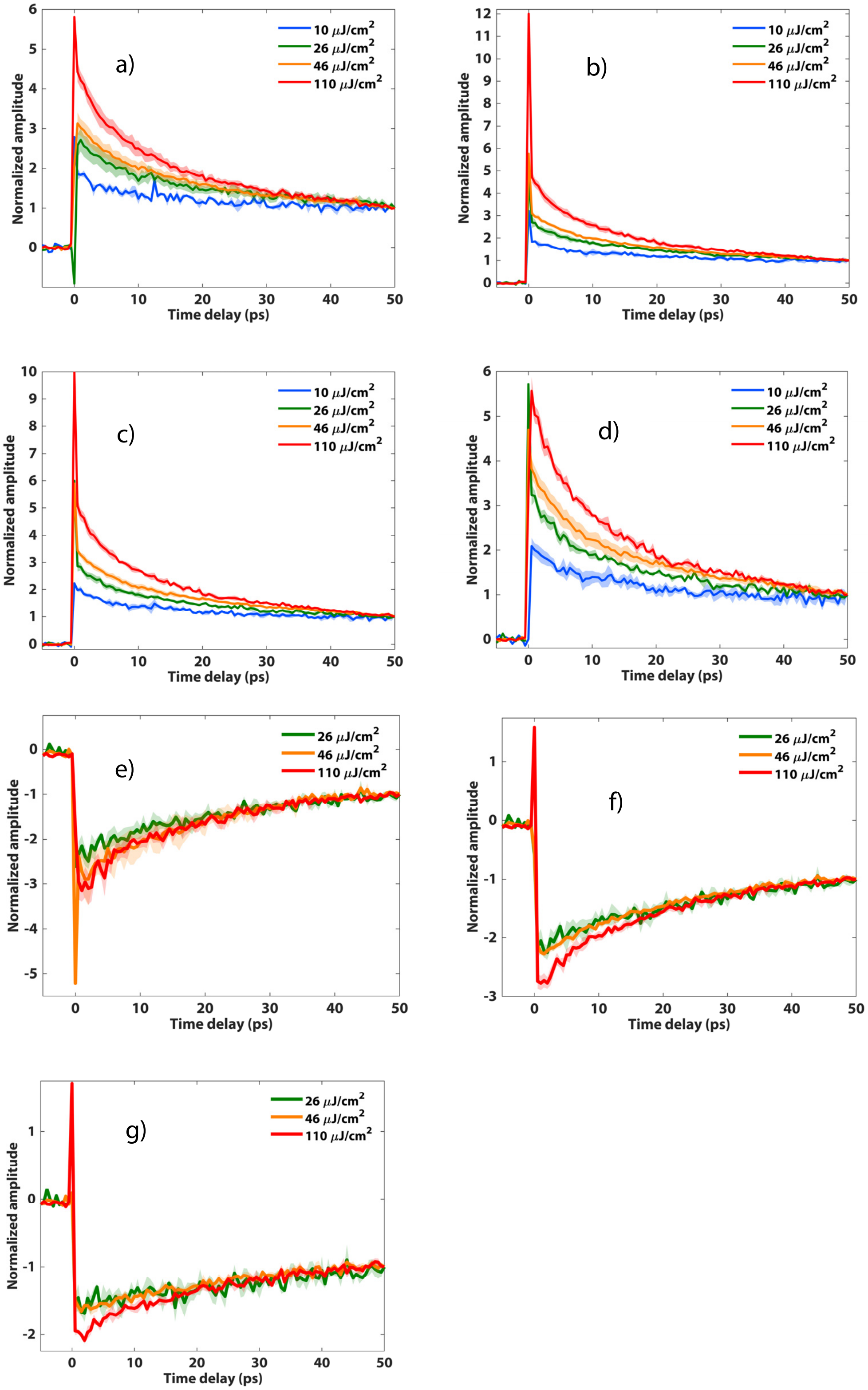

Figure S4: Pump-probe fluence dependent spectral decays plotted at a) 568nm, b) 588nm, c) 596nm and d) 605nm, e) 632nm, f) 642nm, g) 652nm.

Table S3: Fitting constants for biexponential fits of the pump-probe spectral decays at multiple wavelengths:

568nm

	R^2	a_1	T_1 (ps)	a_2	T_2 (ps)
**10 μJ/cm^2**	0.85	0.29	9.28±3.1	0.71	167±15
**26 μJ/cm^2**	0.98	0.42	10.1±1.8	0.58	150±0.76
**46 μJ/cm^2**	0.99	0.40	7.73±0.47	0.60	72±2.6
**110 μJ/cm^2**	0.97	0.49	6.52±0.82	0.51	47±3.5

588nm

	R^2	a_1	T_1 (ps)	a_2	T_2 (ps)
**10 μJ/cm^2**	0.74	0.28	10.0±2.6	0.72	164±14
**26 μJ/cm^2**	0.86	0.33	7.2±1.5	0.67	101±9.7
**46 μJ/cm^2**	0.85	0.36	7.6±0.9	0.64	87±4.3
**110 μJ/cm^2**	0.70	0.47	6.8±0.1	0.53	70±2.0

596nm

	R^2	a_1	T_1 (ps)	a_2	T_2 (ps)
**10 μJ/cm^2**	0.96	0.29	7.2±0.6	0.71	163±13
**26 μJ/cm^2**	0.78	0.35	6.6±0.7	0.65	116±14
**46 μJ/cm^2**	0.87	0.37	7.0±0.7	0.63	94±5.9
**110 μJ/cm^2**	0.87	0.47	6.9±0.5	0.53	82±3.7

605nm

	R^2	a_1	T_1 (ps)	a_2	T_2 (ps)
**10 μJ/cm^2**	0.95	0.35	9.4±2.0	0.65	163±13
**26 μJ/cm^2**	0.89	0.39	6.4±0.9	0.61	123±22
**46 μJ/cm^2**	0.97	0.40	6.9±0.8	0.60	102±16
**110 μJ/cm^2**	0.99	0.49	6.5±0.6	0.51	81±3.7

632nm

	R^2	a_1	T_1 (ps)	a_2	T_2 (ps)
**10 μJ/cm^2**	0.49	0.14	13±7.5	0.86	118±30
**26 μJ/cm^2**	0.93	0.45	21±4.0	0.55	150±12
**46 μJ/cm^2**	0.79	0.99	52±3.8	0.01	174±16
**110 μJ/cm^2**	0.97	0.68	22±3.1	0.32	290±119

642nm

	R^2	a_1	T_1 (ps)	a_2	T_2 (ps)
**10 μJ/cm²**	0.62	0.13	7.9±4.3	0.87	109±23
**26 μJ/cm²**	0.95	0.21	14±2.3	0.79	96±7.3
**46 μJ/cm²**	0.98	0.73	44±3.6	0.27	245±6
**110 μJ/cm²**	0.99	0.52	16±0.4	0.48	147±7.3

652nm

	R^2	a_1	T_1 (ps)	a_2	T_2 (ps)
**10 μJ/cm²**	0.19	0.03	43±41	0.97	216±110
**26 μJ/cm²**	0.75	0.0002	6.8±11	1	127±6.2
**46 μJ/cm²**	0.90	0.0006	24±21	1	130±29
**110 μJ/cm²**	0.97	0.55	37±11	0.45	315±172

Table S4: Comparison of monoexponential and biexponential fits for the photoinduced absorption feature:

Wave-ength	Fluence (μJ/cm²)	Monoexponential R^2	Monoexponential fitting Time	Biexponential R^2 from Table S3
632nm	**10**	0.60	91±8.2	0.49
	**26**	0.92	58±4.0	0.93
	**46**	0.94	53±3.5	0.79
	**110**	0.94	42±3.2	0.97
642nm	**10**	0.70	88±3.7	0.62
	**26**	0.94	65±2.3	0.95
	**46**	0.97	58±2.3	0.98
	**110**	0.96	45±1.1	0.99
652nm	**10**	0.21	219±48	0.19
	**26**	0.77	126±4.1	0.75
	**46**	0.90	130±29	0.90
	**110**	0.95	70±5.1	0.97

We have used biexponential fits based on the compartmental model of Berera and co-workers which attributes multiples DADS with photoinduced absorption signals to the overall dynamics of the wild-type and CK mutants. We expect at least biexponential dynamics based on the overlap of photoinduced absorption and the ground state bleach signatures. We only use biexponential dynamics to show that the changing second decay constant suggests that higher-order exciton effects are at play and exponential dynamics cannot describe the underlying photophysics.

Pump-probe spectra reproducibility

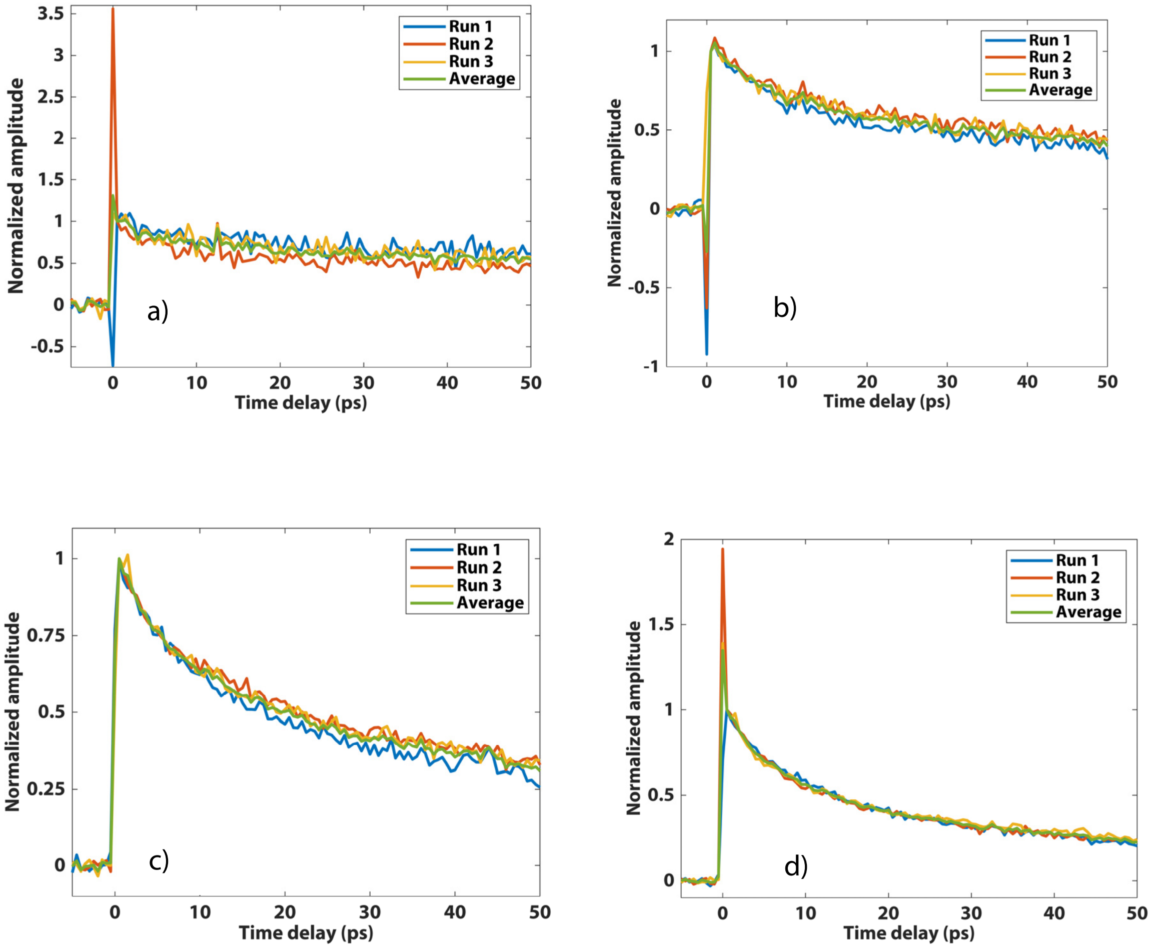

Figure S5: Reproducibility of pump-probe spectra at 568nm for a) 1.3 excitations/rod, b) 3.5 excitations/rod, c) 6.1 excitations/rod and d) 14.6 excitations/rod

Long-time dynamics in PBS

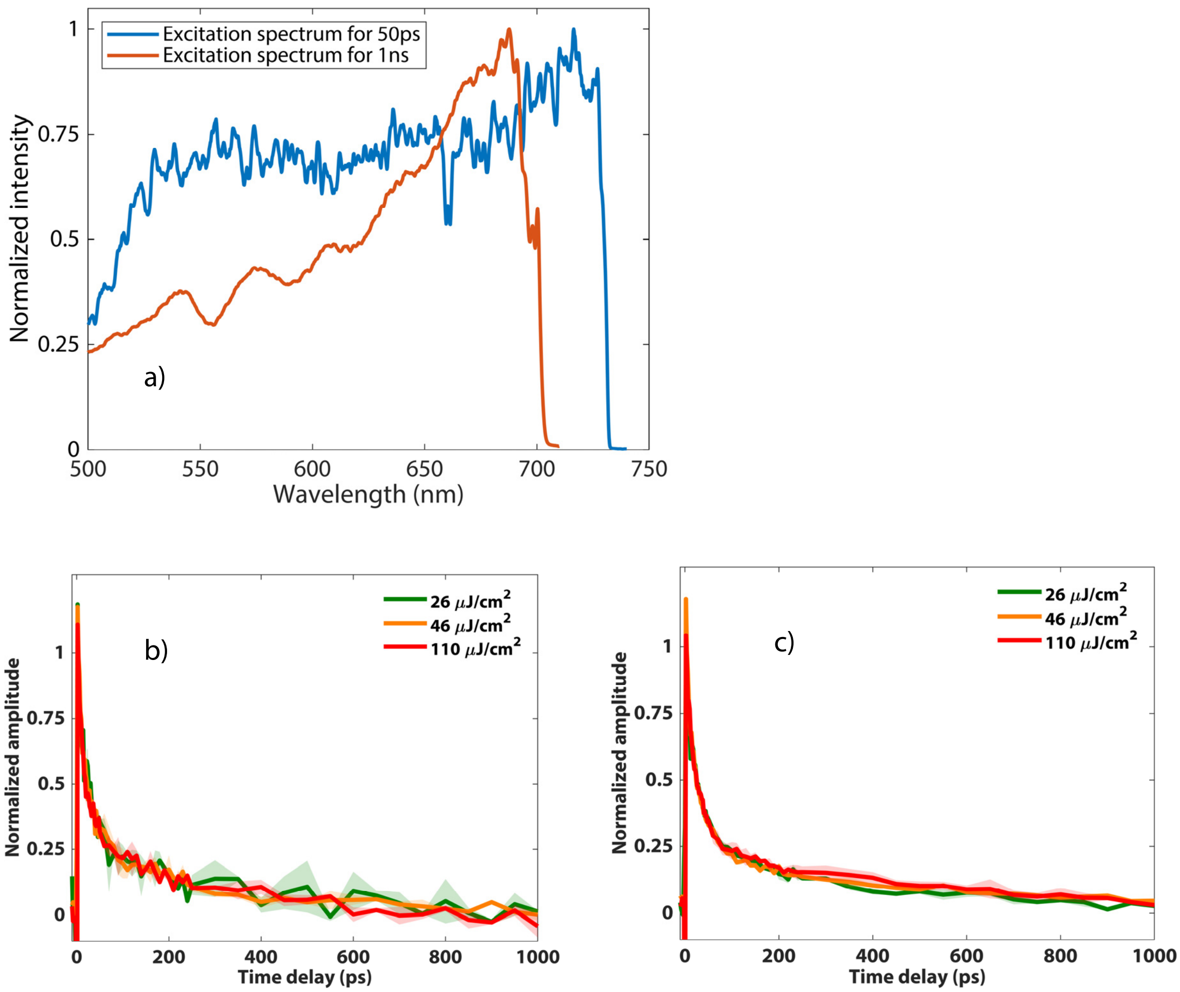

Figure S6: Pump-probe spectra collected up to 1ns delays. a) Excitation spectra for 50ps vs 1ns spectra show the difference in relative excitations at various wavelengths; the overall number of excitations per complex remained the same throughout both measurements. b) 1ns spectra probed at 605nm. c) 1ns spectra probed at 632nm. In b) and c) the relatively low intensity of excitation light at blue wavelengths did not allow for collecting spectra at 568nm.

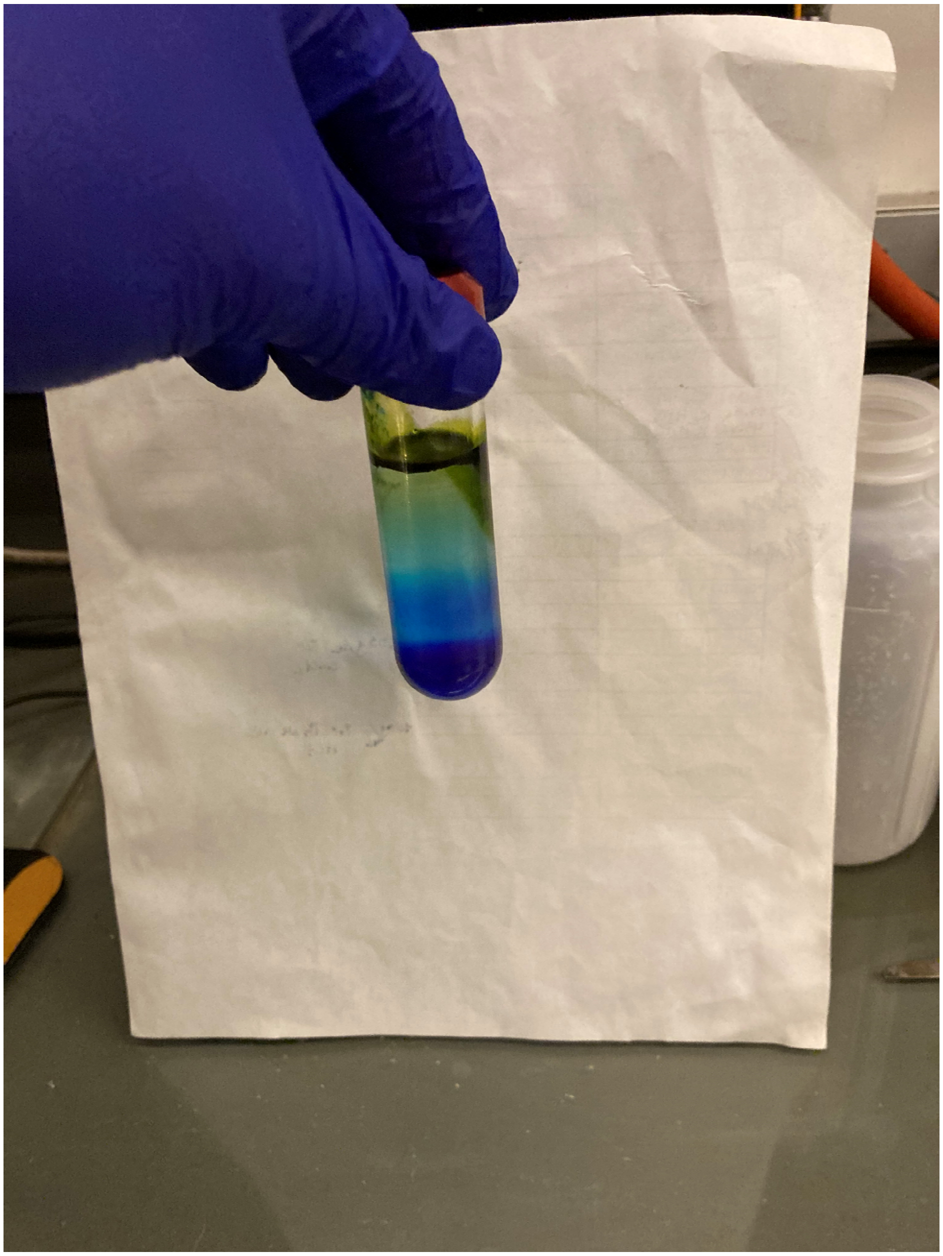

Figure S7: Raw image of discontinuous phycobilisome sucrose gradient with 0.25M, 0.5M, 0.75M and 1.5M sucrose layers in phosphate buffer. Intact phycobilisomes are obtained from the 1.5M layer.

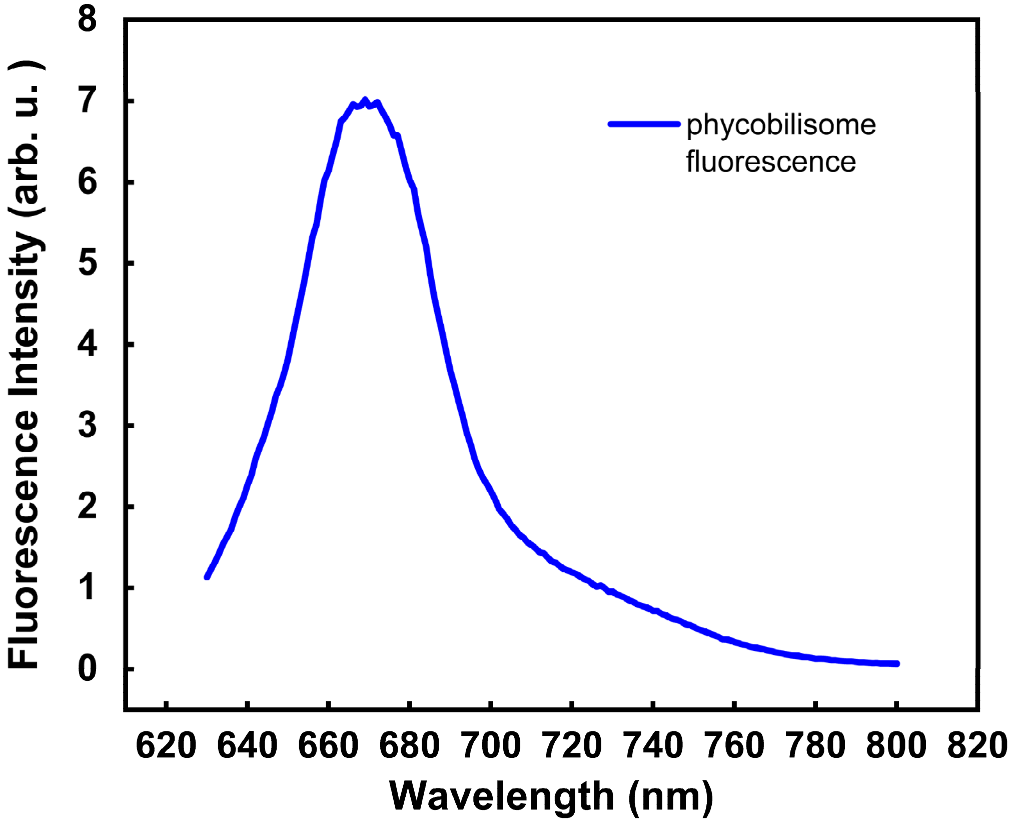

Figure S8: Fluorescence spectrum of the phycobilisome complex at the same concentration as used for pump probe experiments with excitation at 620nm.

Calculation of the frequency of exciton-exciton annihilation in physiological conditions

Our experiment uses pulses with energies of 14 nJ/pulse and a beam diameter or290 µm. 14 nJ/pulse corresponds to 1.3 excitations per PC rod per pulse (see section on calculating the **Number of excitations in pump-probe experiment**).

It is known that excitations move from the PC rods to the APC on the 200ps timescale. However, to be conservative and not overestimate the importance of the observed phenomena, we use an effective lifetime of 100ps in the rods to get the effective power seen by each rod as $\frac{14\,nJ}{0.1\,ns} = 140\,W$.

The effective fluence of the experiment is then $\frac{140\,W}{(290\times10^{-6})^2*\frac{\pi}{4}} = 2.12 \times 10^9\ \frac{W}{m^2}$.

$2.12 \times 10^9\ \frac{W}{m^2}$ corresponds to 1.3 excitations per rod per pulse. If we assume a typical physiological fluence of $7 \times 10^2\ \frac{W}{m^2}$ and that 15% of solar light is absorbed by the phycocyanin rods based on the solar spectrum at the surface of the earth and the absorption cross section, then it corresponds to 6.4×10^{-8} excitations per rod per 100ps.

The Poisson weight for 6.4×10^{-8} number of excitations per rod per 100ps corresponds to 2.04×10^{-15} occurrences of two excitations per 100ps as would be required for annihilation under ambient solar illumination.

Therefore, the number of occurrences of two excitations in one rod simultaneously in fourteen days at 700 W/m^2 with a 12 hour diurnal photoperiod in each rod:

$$2.04 \times 10^{-15}\,\frac{excitations}{100ps} \times 10^{10}\,\frac{100ps}{s} \times 60\ \frac{s}{min} \times 60\,\frac{min}{hour} \times 12\ \frac{hours}{day} \times 14\,days$$
$$\approx \mathbf{13\ occurences}$$

Therefore, at physiological fluences, this phenomenon can occur in each phycocyanin rod ~10 times over a two-week cell lifetime.

References

(1) Sato, T.; Minagawa, S.; Kojima, E.; Okamoto, N.; Nakamoto, H. HtpG, the Prokaryotic Homologue of Hsp90, Stabilizes a Phycobilisome Protein in the Cyanobacterium *Synechococcus Elongatus PCC 7942*: Interaction of HtpG with an in Vivo Substrate. *Mol. Microbiol.* **2010**, *76* (3), 576–589.

(2) Dostál, J.; Fennel, F.; Koch, F.; Herbst, S.; Würthner, F.; Brixner, T., *Nat. Commun.* **2018**, *9* (1), 2466
